# Comparison of enzyme-linked immunosorbent assay systems using rift valley fever virus nucleocapsid protein and inactivated virus as antigens

**DOI:** 10.1186/s12985-018-1071-y

**Published:** 2018-11-22

**Authors:** Fuxun Yu, Ferdinard Adungo, Samson Limbaso Konongoi, Shingo Inoue, Rosemary Sang, Salame Ashur, Allan ole Kwallah, Leo Uchida, Corazon C Buerano, Matilu Mwau, Yan Zha, Yingjie Nie, Kouichi Morita

**Affiliations:** 1 Guizhou Provincial People’s Hospital, Medical College, Guizhou University, No. 83 Zhongshan Dong Road, Guiyang, 550002 Guizhou Province China; 2 Department of Virology, Institute of Tropical Medicine, Nagasaki University, 1-12- 4, Sakamoto, Nagasaki, 852-8523 Japan; 3 Kenya Medical Research Institute, Nairobi, Kenya

**Keywords:** RVFV, Nucleocapsid protein, Expression, IgG sandwich ELISA, IgM capture ELISA

## Abstract

**Background:**

Rift Valley Fever (RVF) is a mosquito-borne viral zoonosis. To detect RVF virus (RVFV) infection, indirect immunoglobulin G (IgG) and immunoglobulin M (IgM) enzyme linked immunosorbent assays (ELISAs) which utilize recombinant RVFV nucleocapsid (RVFV-N) protein as assay antigen, have reportedly been used, however, there is still a need to develop more sensitive and specific methods of detection.

**Methods:**

RVFV-N protein was expressed in *Escherichia coli (E. coli)* and purified by histidine-tag based affinity chromatography. This recombinant RVFV-N (rRVFV-N) protein was then used as antigen to develop an IgG sandwich ELISA and IgM capture ELISAs for human sera. Ninety six serum samples collected from healthy volunteers during the RVF surveillance programme in Kenya in 2013, and 93 serum samples collected from RVF-suspected patients during the 2006–2007 RVF outbreak in Kenya were used respectively, to evaluate the newly established rRVFV-N protein-based IgG sandwich ELISA and IgM capture ELISA systems in comparison with the inactivated virus-based ELISA systems.

**Results:**

rRVFV-N protein-based-IgG sandwich ELISA and IgM capture ELISA for human sera were established. Both the new ELISA systems were in 100% concordance with the inactivated virus-based ELISA systems, with a sensitivity and specificity of 100%.

**Conclusions:**

Recombinant RVFV-N is a safe and affordable antigen for RVF diagnosis. Our rRVFV-N-based ELISA systems are safe and reliable tools for diagnosis of RVFV infection in humans and especially useful in large-scale epidemiological investigation and for application in developing countries.

## Background

Rift Valley Fever (RVF) is a mosquito-borne viral zoonosis, which periodically causes disease outbreaks in humans and livestock. Infection by RVF virus (RVFV) causes abortion or resorption of the fetus in pregnant domestic ruminants, with newborn mortality approaching 100%. Transmission of RVFV to humans, either through contact with bodily fluids of infected animals or through mosquito bites, may result in mild to moderate influenza-like symptoms and severe retinitis, encephalitis and hemorrhagic fever [1, 2].

RVFV was first described in Kenya in 1931 [3]. Major outbreaks of RVF have been reported in Egypt in 1977, Kenya in 1997–1998, Saudi Arabia in 2000–2001, Yemen in 2000–2001, and South Africa in 1951,1974–1976 and 2010 [1, 4–8]. The recent outbreak of RVF in Kenya in November 2006 through March 2007, where several hundred human cases were confirmed in 6 of 8 provinces had a devastatingly high case-fatality rate for hospitalized patients while there were up to 180,000 infected mildly ill or asymptomatic people within the highly affected areas [9–11]. Sero-surveys suggested an attack rate up to 13% of residents in heavily affected areas; livestock deaths and abortions were also noted during the outbreak [9, 11].

RVFV belongs to the order of *Bunyavirales,* family *Phenuiviridae*, genus *Phlebovirus*. The members of this family carry a tri-segmented single stranded RNA genome. The M (medium) and L (large) segments are of negative orientation, whereas the S (small) segment has an ambisense polarity. The L segment encodes the RNA-dependent RNA-polymerase while the M segment encodes the two glycoproteins Gn and Gc and also the non-structural protein NSm. The small segment encodes the nucleocapsid (N) protein and a non-structural protein NSs using an ambisense coding strategy [1, 2]. The N protein is highly conserved and it is one of the most immunodominant viral proteins among members of the *Phenuiviridae* family [12–14].

ELISA systems based on inactivated antigens derived from tissue culture or mouse brain have been validated for RVF serodiagnosis in humans and animals [15–18]. The high production costs of the antigen and the requirement of a high biocontainment facility limited its application. rRVFV-N-based ELISA systems have been reported in the diagnosis of RVFV infection in humans and animals [19–24]. Most of the rRVFV-N-based ELISA systems in humans are used in indirect IgG and indirect IgM methods. There are no reports of rRVFV-N-based IgG-sandwich and IgM capture ELISAs for human serum. For human serodiagnosis, the application of rRVFV-N protein is limited and the whole virus-based ELISA systems are still recommended by WHO for RVF diagnosis.

To evaluate the possibility of using rRVFV-N protein as safe and affordable antigen for serodiagnosis of RVFV infection, we cloned the full length RVFV N gene, expressed the N protein in *E.coli*, purified the N protein and evaluated the rRVFV-N-based ELISA systems in comparison with the existing inactivated RVFV-based ELISA systems.

## Methods

### Serum samples

Ninety six serum samples collected from healthy volunteers in 2013 during an RVF surveillance study in Kenya and 93 serum samples collected from suspected-RVF patients during the 2006–2007 RVF outbreak in Kenya were used for the experiments on the detection of IgG and IgM against RVFV, respectively.

### Construction of recombinant plasmids

Wild type RVFV isolate 46A-66, isolated from mosquitoes that were collected during the 2006 RVF outbreak in Kenya, was used to construct RVFV recombinant plasmids. The wild type virus was cultured at the Kenya Medical Research Institute for more than 5 passages. RNAs from RVFV infected culture fluid of Vero cells were extracted by using the QIAamp viral mini kit (Qiagen, Hilden, Germany) according to the manufacturer’s instructions. The RVFV N gene was amplified by RT-PCR using the sense primer 5′- AACGGATCCACAATAATGGACAACTAT-3′ and the reverse primer 5’-CCTGGTCGACCACTTAGGCTGCTGT-3′. The sense and reverse primers contained the Bam HI and Sal I restriction sites (underlined nucleotides), respectively. The PCR amplified DNA fragment was digested with Bam HI and Sal I, purified by a QIAEX II gel extraction kit (Qiagen, Hilden, Germany), and subsequently cloned into the corresponding restriction site of the pQE30 vector (Qiagen, Hilden, Germany). The insert of recombinant plasmid was confirmed to be in frame by DNA sequencing. The expression construct, encompassing the coding region for amino acids (aa) 1–245, the full length of RVFV-N protein with a vector derived His-tag (histidine hexamer) at the N-terminus was obtained. The resultant recombinant protein was designated as recombinant RVFV-N protein.

### Expression and purification of the rRVFV- N protein

The rRVFV-N protein was expressed by inserting the recombinant plasmid containing the RVFV-N sequence into *E. coli* strain XL-1 blue. The expression and purification was done as described previously [25]. Briefly *E. coli* strain *XL-1 blue* containing the rRVFV-N recombinant plasmid was cultured at 37 °C in 1 litre of Luria-Bertani (LB) medium containing 100 μg/ml of ampicillin and then induced for 3 hrs by 0.2 mM isopropyl β-D-thiogalactoside (IPTG) when the optical density (OD 600 nm) reached 1.0. After harvest by centrifugation at 4 °C for 30 min, the *E. coli* pellet was resuspended in 10 mM PBS at pH 7.5 with 500 mM NaCl and frozen at − 80 °C. After freezing and thawing three times, the cell suspension was sonicated for 2 min with an interval of 1 s between pulses and centrifuged at 30,000 g for 30 min at 4 °C. The supernatant was then applied to a Talon™ IMAC resin column (Clontech, USA) and the rRVFV-N protein was purified according to the manufacturer’s instructions. Protein concentrations were determined by the Bradford method using a Bio-Rad protein assay reagent kit (Bio-Rad, USA), and the purity of the protein was analyzed by sodium dodecyl sulfate-polyacrylamide gel electrophoresis (SDS-PAGE).

### Western blot analysis

Western blot analysis was performed as described previously [26]. Briefly, the recombinant protein was separated by SDS-PAGE and then transferred onto a PVDF membrane (Immobilon, Millipore, USA). After blocking with Blockace (Yokijirushi, Sapporo, Japan) overnight at 4 °C, the membrane was subjected to reaction with rabbit RVFV hyper-immune sera (1:400) or mouse anti-histidine (1:1000 dilution) for 1 hr at 37 °C, followed by incubation with horseradish peroxidase conjugated- goat anti-rabbit IgG, or rabbit anti-mouse IgG (1:1000 dilution) for 1 hr at 37 °C. Finally, the reaction was visualized by dimethyl amino benzidine (DAB) staining.

### Preparation of rabbit hyper-immune sera for RVFV

Two litres of infected culture fluid (ICF) was harvested from cultures of Vero cells 5 days after inoculation with RVFV (Smithburn strain). ICF was purified by sucrose-gradient ultracentrifugation at 50,000 x g for 14 hrs at 4 °C. The purified virus (0.25 mg/mL) was inactivated with 1% (final concentration) formalin for about 2 days at 4 °C. Two 3-month-old New Zealand white rabbits were immunized once with formalin-inactivated virus mixed with an equal volume of Freund’s complete adjuvant (0.125 mg/shot/rabbit), and after 1 week boosted thrice with formailin-inactivated virus mixed with an equal volume of Freund’s incomplete adjuvant at intervals of 1 week. Serum was collected and the rabbit anti-virus IgG was purified by using MAb Trap Kit (GE Healthcare) according to the manufacturer’s instructions.

### Preparation of recombinant severe fever with thrombocytopenia syndrome virus nucleocapsid (rSFTSV-N) protein

The rSFTSV-N protein was expressed by inserting the recombinant plasmid containing the N gene sequence of severe with thrombocytopenia syndrome virus (a newly emerged *Phlebovirus* in east Asia) into *E. coli* strain XL-1 blue, and purified following the procedure described previously [25]. This protein was used as negative antigen in our newly developed ELISA systems.

### ELISA procedures

To evaluate the possibility of applying the rRVFV-N protein for diagnosis of RVFV infection, we developed an IgG sandwich and an IgM capture ELISA system for human sera by using rRVFV-N protein as antigen. The results were compared with those of inactivated virus-based ELISA systems routinely used in the WHO RVF reference diagnostic laboratories.

For all the ELISA procedures, the plates used were 96-well Nunc immunoplates (Thermo Scientific, Denmark), and all the reagents were in 100 μL volumes. Wash buffer was 0.01 M PBS with 0.1% (vol/vol) Tween 20 (PBS-T). Plates were washed thrice with PBS-T between reagent steps. The coating buffer was 0.01 M PBS, pH 7.4, and coated plate was left at 4 °C overnight. All subsequent serum samples and reagent dilutions were done in PBS-T with 5% nonfat milk (Difco, Detroit, USA). Incubations, except for substrate, were done for 1 hr at 37 °C. Plates added with H_2_O_2_-ABTS substrate (Kirkegaard & Perry, Gaithersburg, MD) were incubated for 30 min at 37 °C before reading spectrophotometrically. On each plate, 3 control serum samples namely positive control, weak positive control and negative control were added. Optical density (OD) values at 410 nm were recorded. The adjusted OD was calculated by subtracting the OD of the negative antigen reacted wells from the OD of the positive antigen reacted wells. The OD cut-off value was calculated as the mean of the adjusted OD of the negative control sera plus three times the standard deviation, and generally this would give an OD of ≤0.2 at a 1:100 sample dilution. A serum sample was considered positive if the adjusted OD value was greater than or equal to the assay cut-off or 0.2, which ever value was higher.

### IgG sandwich ELISA using inactivated RVFV

The IgG sandwich ELISA system using inactivated RVFV was routinely used in the WHO RVF diagnosis reference laboratory in the Kenya Medical Research Institute. The virus strain used in the inactivated virus-based assay systems was ZH501 strain (GenBank: DQ380149.1). The virus was propagated in Vero cells and the infected culture fluid (supernatants) from these cells provided the RVFV antigens used in the assay. The supernatants were inactivated by using 0.3% beta-propiolactone and cobalt irradiated using 3 million rads and were tested for safety using established protocols [27, 28]. Briefly, for each sample to be tested, 4 wells were subjected to the following reaction steps: coating with anti-RVFV hyper-immune mouse ascetic fluid (HMAF) diluted at 1:8000; then, addition of 1:18 dilution of inactivated RVFV culture fluid (positive antigen) to two of these wells and to the other two wells with 1:18 dilution of mock culture fluid (negative antigen); followed by the addition of test serum samples (diluted 1:100) to all the 4 wells; and then, detection of bound IgG by 1:8000 diluted peroxidase-conjugated mouse anti-human IgG (Fc specific) (Accurate Chemical & Scientific Corp, Westbury, NY) after which the ABTS substrate was added to produce a color reaction for OD detection.

In our initial experiments, the procedure above was applied to 12 serum samples and the results obtained were compared with the results when only the inactivated virus antigen in the inacivated virus based sandwich ELISA system was replaced with the rRVF-N (50 ng/well) we produced. This comparison was done to determine the reactivity of rRVF-N in the inactivated virus-based ELISA system used in the WHO reference laboratory in Kenya.

### IgG sandwich ELISA using recombinant RVFV-N

The procedure that we developed for IgG sandwich ELISA utilizing rRVFV-N followed the same steps of inactivated virus-based ELISA procedure with some modifications.

Briefly, for each serum sample, 4 wells were subjected to the following reaction steps: coating with 1:10,000 dilution of the purified rabbit RVFV hyper-immune serum IgG prepared as mentioned above, addition of 50 ng rRVFV-N protein (positive antigen) to two wells and of 50 ng rSFTSV-N protein (negative antigen) to the other two wells, followed by the addition of 1:100 diluted human serum sample to the four wells, then addition of 1:30,000 diluted horseradish-peroxidase-conjugated goat anti-human IgG (American Qualex, Califonia, USA); and lastly, color development was done after the addition of ABTS substrate.

The optimal concentration of capture antibody (purified rabbit RVFV hyper-immune serum IgG) used to coat the plate and the rRVFV-N protein was determined by checkerboard titration with two positive and two negative reference serum samples.

### IgM capture ELISA using inactivated RVFV

The IgM capture ELISA system using inactivated RVFV was routinely used for diagnosis in the WHO RVF diagnosis reference laboratory in the Kenya Medical Research Institute. Briefly, for each sample to be tested, 4 wells were coated with 1:500 dilution of anti-human IgM (Kirkegaard & Perry Laboratories) after which 1:100 diluted human serum sample was added to all four wells. Two wells were added with positive antigen (inactivated RVFV culture fluid, which was also the same source of antigen in our IgG sandwich ELISA) and the other two wells with negative antigen (mock culture fluid). The wells were then reacted with 1:2000 anti-RVFV HMAF, which were then reacted with 1:16,000 diluted HRP conjugated anti-mouse IgG (Kirkegaard & Perry Laboratories) and color development was done by adding ABTS substrate.

Similar to the IgG sandwich ELISA, to determine the reactivity of rRVFV-N in the inactivated virus-based IgM capture ELISA system, 12 serum samples were tested using the described procedure above with the inactivated RVFV antigen and with our alternative rRVF-N (50 ng/well).

### IgM capture ELISA using rRVFV-N protein

The rRVFV-N-based IgM capture ELISA followed the same steps as with the inactivated virus-based ELISA system with some modifications. For each serum sample to be tested, 4 wells were coated with 1:500 dilution of anti-human IgM (Cappel, MP Biochemicals). Then 1:100 diluted human serum was added to the 4 wells. Two wells were added with 50 ng rRVFV-N protein (positive antigen) and the other two wells were added with 50 ng of rSFTSV-N protein (negative antigen). A 1:10,000 dilution of anti-RVFV rabbit hyperimmune serum was added to all four wells, after which a 1:10,000 diluted peroxidase conjugated anti-rabbit IgG (American Qualex, Califonia, USA) was then added. Color development was done by adding ABTS substrate.

## Results

### Expression and purification of rRVFV-N protein

**Fig. 1:**
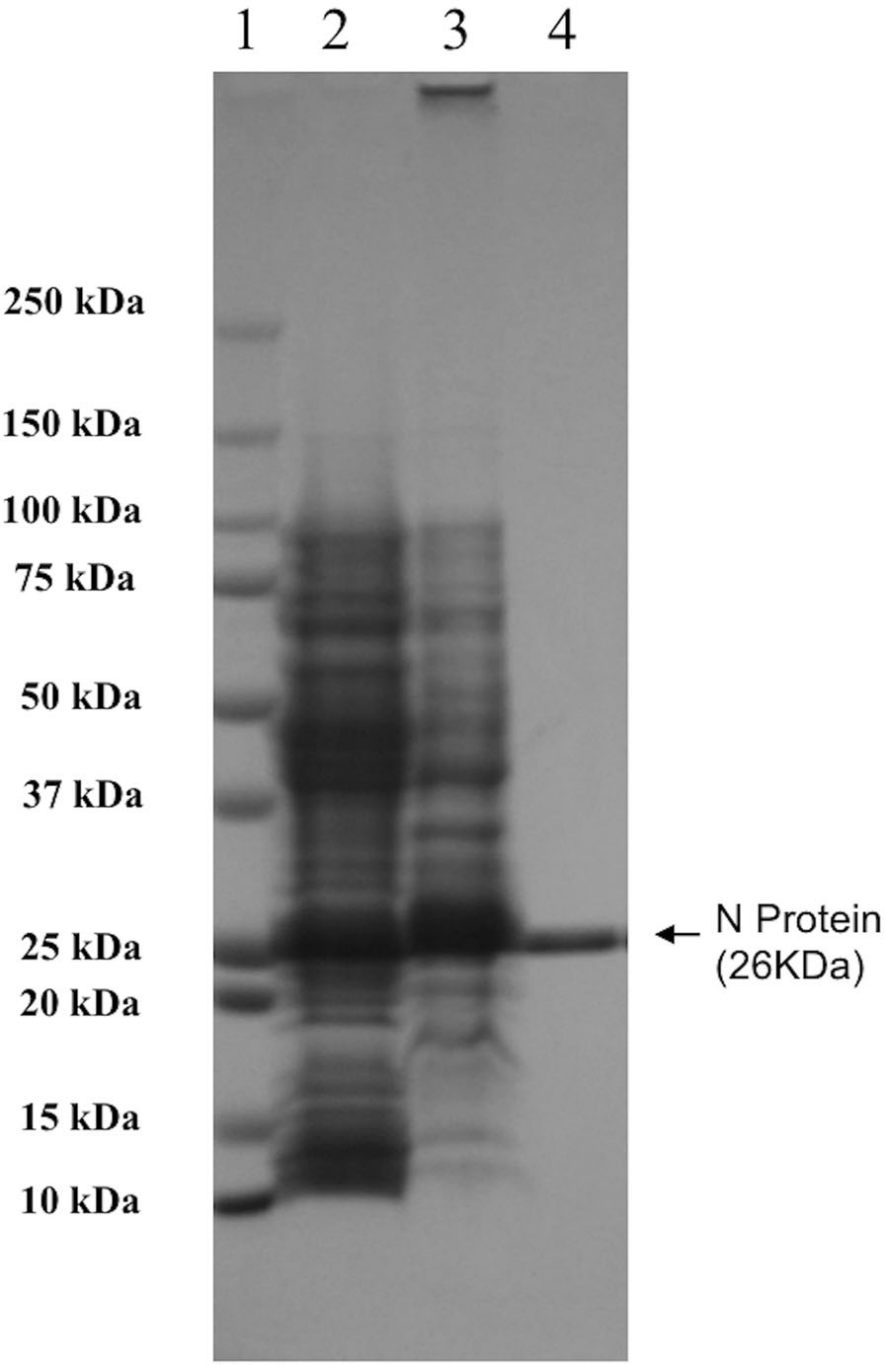
Expression and purification of recombinant RVFV-N protein. Recombinant plasmid containing the full length RVFV N gene was transformed into *E. coli* XL1-blue strain and induced with IPTG. *E.coli* cells were collected and dissolved in 10 mM PBS (pH 7.5) with 500 mM NaCl. After sonication, the *E. coli* cell lysate was centrifuged and the recombinant protein was purified from the supernatant by Talon™ IMAC affinity column. The *E. coli* cell lysate and purified recombinant protein were analyzed in a 10% SDS-PAGE gel and revealed with Coomassie brilliant blue staining. Lane 1: protein marker (Precision plus protein standards, Bio-Rad); Lane 2: supernatant of sonicated *E. coli* cell lysate after centrifugation; Lane 3: pellet of sonicated *E.coli* cell lysate; Lane 4: purified recombinant protein

**Fig. 2:**
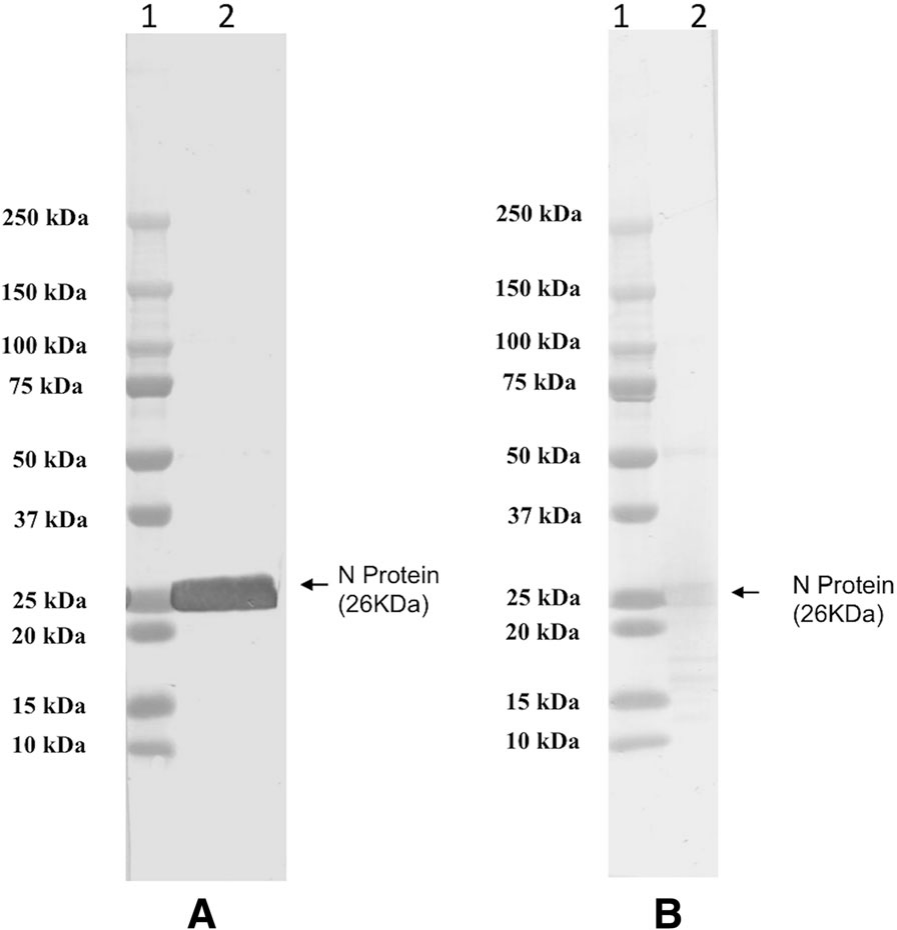
Western-blot analysis of purified RVFV-N protein. Pre-stained protein marker and purified recombinant RVFV-N protein were separated by SDS-PAGE and transferred to a PVDF membrane. Each membrane was incubated with diluted rabbit RVF hyper-immune sera or mouse anti-Histidine serum followed by horseradish peroxidase(HRP)-conjugated anti-rabbit IgG or anti-mouse IgG (1:1000 dilution) and detected by DAB staining. **a** Reactivity of recombinant protein to rabbit RVF hyper-immune sera. **b** Reactivity of recombinant protein to mouse anti-Histidine antibody. Lane 1: protein marker (Precision plus protein standards, Bio-Rad); Lane 2: purified RVFV-N protein

The RVFV nucleocapsid gene, encompassing the codons of amino acid residues 1–245 of the full length RVFV-N protein, was successfully expressed in *E. coli* mostly in soluble form. The sequence and reading frame of the N gene were confirmed by sequencing the DNA of the recombinant plasmid. This rRVFV-N protein was purified from the supernatant by Talon metal affinity column under non-denaturing conditions. After SDS-PAGE, Coomassie blue staining revealed a single protein band of 26 kDa as predicted (Fig. 1). The identity of the rRVFV-N protein was further confirmed by Western blot with RVFV rabbit hyper immune serum and mouse antibody to histidine-tag (Fig. 2).

### Reactivity of recombinant RVFV-N protein in the inactivated virus-based IgG sandwich and IgM capture ELISA systems

**Fig. 3:**
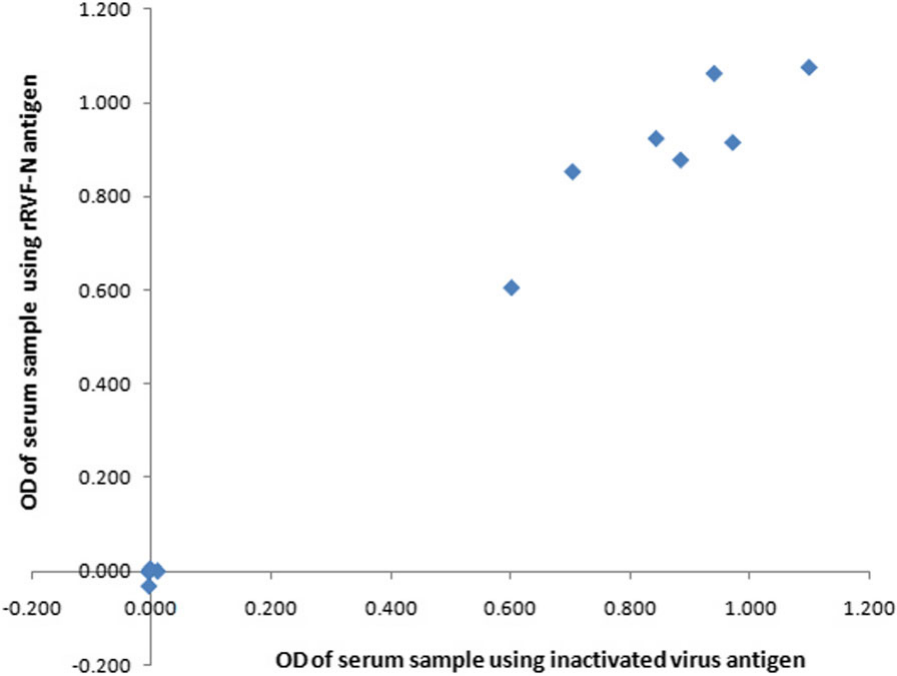
Comparison of rRVFV-N protein with inactivated RVFV antigen in inactivated virus based-IgG sandwich ELISA system. Inactivated virus-based IgG sandwich ELISA system used in the RVF diagnosis reference laboratory was used to evaluate the rRVFV-N protein by replacing the inactivated virus antigen (RVFV culture fluid) with the rRVFV-N protein. Twelve human serum samples collected during the RVF surveillance were tested by using the virus culture fluid (X axis) or rRVFV-N protein as antigen (Y axis)

**Fig. 4:**
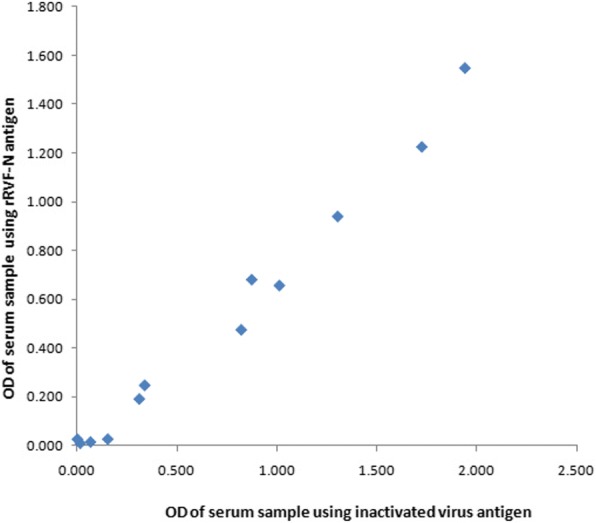
Comparison of rRVFV-N protein with inactivated virus antigen in inactivated virus based-IgM capture ELISA system. Inactivated virus based IgM capture system used in the RVF diagnosis reference laboratory was used to evaluate the RVFV-N protein by replacing the inactivated virus antigen (RVFV culture fluid) with the rRVFV-N protein. Twelve human serum samples collected from an RVF outbreak were tested by using the virus culture fluid (X axis) or rRVFV-N protein as antigen (Y axis)

The 12 serum samples collected from human volunteers during an RVF surveillance had OD values in the inactivated virus-based IgG sandwich ELISA systems comparable to the values when the inactivated virus antigen was replaced with our rRVFV-N antigen (Fig. 3). Similar OD values were also observed in the other 12 human serum samples collected during an RVF outbreak in the inactivated virus-based IgM capture ELISA system with inactivated virus antigen and with our rRVFV-N- antigen were applied (Fig. 4).

### Evaluation of rRVFV-N protein-based IgG sandwich ELISA

Among the 96 human serum samples (including the 12 samples tested above) collected during the RVF surveillance programme in Kenya, 11 samples were IgG positive and 85 were IgG negative by our IgG sandwich ELISA system. These results perfectly matched the inactivated virus-based IgG sandwich ELISA results. The sensitivity and specificity of our system were 100% and with a 100% concordance to the inactivated virus based ELISA system (Table 1).

**Table 1:** Comparison of RVFV-N-based IgG sandwich ELISA with inactivated virus-based IgG sandwich ELISA for human serum

Inactivated virus-based IgG ELISA	rRVFV-N IgG ELISA		Total
	Positive	Negative	
Positive	11	0	11
Negative	0	85	85
Total	11	85	96
Concordance^a^: 100%	Sensitivity^b^: 100%		Specificity^c^: 100%

^a^(No. of samples positive by both methods + No. of samples negative by both methods)/total number of samples × 100

^b^True positive/(true positive + false negative) × 100

^c^True negative/(true negative + false positive) × 100

### Evaluation of recombinant RVFV-N protein based IgM capture ELISA

Among 93 human serum samples (including the 12 samples tested above) collected from RVF-suspected patients during the 2006–2007 outbreak in Kenya, 42 samples were IgM positive and 51 were IgM negative by IgM capture ELISA using our new IgM capture ELISA system. These results perfectly matched the inactivated virus based IgM capture ELISA results. Our system had a sensitivity and specificity of 100% and with a 100% concordance to the inactivated virus based IgM ELISA system (Table 2).

**Table 2:** Comparison of RVFV-N-based IgM capture ELISA with inactivated virus-based IgM capture ELISA for human serum

inactivated virus-based IgM ELISA	rRVFV-N IgM ELISA		Total
	Positive	Negative	
Positive	42	0	42
Negative	0	51	51
total	42	51	93
Concordance^a^: 100%	Sensitivityb: 100%		Specificity^c^: 100%

^a^(No. of samples positive by both methods + No. of samples negative by both methods)/total number of samples × 100

^b^True positive/(true positive + false negative) × 100

^c^True negative/(true negative + false positive) × 100

## Discussion

We have successfully expressed rRVFV-N protein in *E. coli* and purified the protein to near homogeneity by his-tag based affinity chromatography under native conditions (Fig. 1). The purified rRVFV-N protein reacted with RVFV rabbit hyper immune serum and mouse anti-histidine antibody in Western blot (Fig. 2). Most of the expressed rRVFV-N protein was in the soluble form and the purification process was done under native condition without using any detergent, and thereby omitting the tedious process of refolding a denatured protein. The expression and purification procedures described in this study provide a simple and efficient way to obtain pure rRVFV-N protein in large quantity.

Recombinant RVFV N protein based ELISA systems have been reported in the diagnosis of RFV infection in humans and animals [19–24]. Fafetine et al., reported indirect IgG and indirect IgM ELISA systems for domestic sheep, goats and cattle [19] and compared a recombinant N protein based indirect IgG ELISA with an IgG sandwich ELISA using sera collected from small domestic ruminants [24]. Jansen van Vuren et al. reported indirect IgG and indirect IgM ELISA system for humans and sheep using recombinant RVFV N protein as coating antigen [20]. Paweska et al. evaluated recombinant N protein based indirect IgG ELISA for humans and African buffalos [21, 22]. Williams et al developed an IgM capture ELISA for domestic animals [23]. In all these reports, rRVFV-N protein works well as an antigen, proving the usefulness of rRVFV-N protein. But most of the reports have used indirect IgG and IgM ELISA systems where the coating reagent is the assay antigen, which in turn is allowed to react with the test serum supposedly containing the primary antibody followed by an enzyme-labeled secondary antibody. Recombinant RVFV-N protein-based IgG sandwich ELISA and IgM capture ELISA systems for humans have not been reported. Unlike the indirect ELISAs, the IgG sandwich and IgM capture ELISAs have antibodies as coating reagents to capture the assay antigen and the IgM from test sera, respectively. It has been shown in small ruminants that IgG sandwich ELISA is more sensitive than IgG indirect ELISA [24]. For human diagnosis, indirect IgM ELISA suffers from potential false-positive results because of the existence of rheumatoid factor in some individuals and from false-negative results because of the competition from the IgG antibody [29]. IgM capture ELISA is recommended for more sensitive and more specific detection.

Our evaluation on the reactivity of the rRVFV-N protein in the inactivated virus-based IgG sandwich ELISA and IgM capture ELISA using 12 human serum samples for each ELISA system showed that among the 12 samples tested for IgG (Fig. 3) and another 12 samples for IgM (Fig. 4), the results using the rRVFV-N protein perfectly matched with those using the inactivated virus antigen. This indicates that N protein is an important immunoreactive component of the RVFV.

Using the rRVFV-N protein, we then developed our own IgG sandwich ELISA and IgM capture ELISA for human serum and compared with the inactivated virus-based ELISA systems. After applying them to 96 healthy human serum samples collected during the RVF surveillance programme in Kenya or to 93 serum samples collected from RVF-suspected patients during the 2006–2007 RVF outbreak, we showed that the rRVFV-N protein-based IgG sandwich ELISA (Table 1) and IgM capture ELISA (Table 2) were in 100% concordance to the inactivated virus-based ELISA systems with a sensitivity and specificity of 100%. These findings clearly demonstrated the usefulness of the rRVFV-N protein-based ELISA systems for reliable clinical diagnosis of RVFV infection in humans.

Compared to the inactivated virus based-ELISAs, which were used by most researchers for the RVF diagnosis, the rRVFV-N protein-based ELISAs used in this study offer several distinct advantages such as the elimination of the use of infectious virus in the antigen production, the easier way to standardize the test because variable factors (virus multiplicity of infection, virus strains, cell line, cell condition) associated with the preparation of inactivated virus are no longer present, the short period of time (within 1 week after cloning) and the low costs required for the preparation of the recombinant antigen. It would be especially useful in cases of large-scale epidemiological investigation and for application in developing countries.

## Conclusions

Recombinant RVFV-N is a safe and affordable antigen for RVF diagnosis. However, the current evaluation used a small number of samples thus, further in-depth validation of the assays are required before these could be used to replace the currently accepted assays based on whole antigen. The newly established rRVFV-N-based IgG sandwich and IgM capture ELISA systems may offer safe and reliable tools for diagnosis of RVFV infection in humans and are especially useful in large-scale epidemiological investigation and for application in developing countries.

## Abbreviations

DAB: Dimethyl amino benzidine
*E. coli*: *Escherichia coli*
ELISA: Enzyme linked immunosorbent assay
ICF: Infected culture fluid
IgG: Immunoglobulin G
IgM: Immunoglobulin M
OD: Optical density
PBS-T: 0.01 M PBS with 0.1% (vol/vol) Tween 20
rRVFV-N: Recombinant RVFV-N
RVF: Rift valley fever
RVFV-N: Rift valley fever virus nucleocapsid
